# [68Ga]Ga-DOTANOC Uptake at Pancreatic Head/Uncinate Process: Is It a Persistent Diagnostic Pitfall Over Time?

**DOI:** 10.3390/cancers14143541

**Published:** 2022-07-21

**Authors:** Elena Tabacchi, Emilia Fortunati, Giulia Argalia, Lucia Zanoni, Diletta Calabrò, Silvi Telo, Davide Campana, Giuseppe Lamberti, Claudio Ricci, Riccardo Casadei, Stefano Fanti, Valentina Ambrosini

**Affiliations:** 1 Nuclear Medicine, IRCCS, Azienda Ospedaliero-Universitaria di Bologna, 40138 Bologna, Italy; elena.tabacchi@aosp.bo.it (E.T.); lucia.zanoni@aosp.bo.it (L.Z.); s.fanti@unibo.it (S.F.); valentina.ambrosini@unibo.it (V.A.); 2 Nuclear Medicine, Alma Mater Studiorum, University of Bologna, 40138 Bologna, Italy; giulia.argalia@studio.unibo.it (G.A.); dilecala@gmail.com (D.C.); silvi.telo@gmail.com (S.T.); 3 Nuclear Medicine Unit, Ospedale Guglielmo da Saliceto, Azienda USL di Piacenza, 29121 Piacenza, Italy; 4 Nuclear Medicine Unit, AUSL Romagna, 47521 Cesena, Italy; 5 Oncology, IRCCS, Azienda Ospedaliero-Universitaria di Bologna, 40138 Bologna, Italy; davide.campana@unibo.it; 6 DIMES, Alma Mater Studiorum, University of Bologna, 40138 Bologna, Italy; giuseppe.lamberti8@unibo.it; 7 Pancreatic Surgery, IRCCS, Azienda Ospedaliero-Universitaria di Bologna, 40138 Bologna, Italy; claudio.ricci6@unibo.it (C.R.); riccardo.casadei@aosp.bo.it (R.C.); 8 DIMEC, Alma Mater Studiorum, University of Bologna, 40138 Bologna, Italy

**Keywords:** [68Ga]Ga-DOTANOC, PET/CT, neuroendocrine tumors, head pancreas, uncinate process

## Abstract

**Simple Summary:**

The purpose of the present study is to describe the frequency of non-malignant [68Ga]Ga-DOTANOC uncinate process (UP) uptake and to evaluate its variations over time. Among the first 6 months of enrolment of a monocentric prospective observational electronic archive, analyses of a large number of PET/CT scans (n = 268) of NEN pts (n = 172) demonstrated that: UP uptake is a frequent finding (observed in almost half of the cases, slightly higher than previously reported), mostly presenting with a diffuse pattern and, interestingly, characterized by variations over time in almost one third of the cases. The consciousness of this diagnostic pitfall is of utmost importance for accurate [68Ga]Ga-DOTANOC PET/CT reporting, since the pancreas represents one of the most frequent sites of NEN.

**Abstract:**

Purpose: [68Ga]Ga-DOTA-peptide uptake in the pancreatic head/uncinate process (UP) is a frequent PET/CT finding. Although mostly physiologic, it can represent a pitfall in PET/CT reading, especially when focal. An increased frequency of UP uptake has been reported in patients (pts) affected by diabetes mellitus (DM). The aim of the study is to describe the frequency of [68Ga]Ga-DOTANOC UP uptake to evaluate its variations over time and its possible correlation with DM. Methods: In September 2017, a monocentric prospective observational electronic archive was initiated at our center to collect clinical and imaging data of pts undergoing [68Ga]Ga-DOTANOC PET/CT. Among the pts enrolled in the first 6 months (Sept 2017 to Feb 2018), those presenting [68Ga]Ga-DOTANOC PET/CT uptake at UP level were included. Pts with UP lesions already documented on CT/MRI or those that underwent surgical excision of UP before PET/CT were excluded from the analysis. [68Ga]Ga-DOTANOC UP uptake was classified as diffuse or focal and compared with the pattern observed in previous PET/CT scans performed at our center. An increased frequency of UP uptake was also correlated with the presence of DM. Results: In the first 6 months, 253 pts were enrolled in the archive and 172 out of them were included in the analysis. UP increased uptake was frequently observed (77/172, 44.8%) and was mostly diffuse (62/77). In 75/172 pts (43.6%), previous [68Ga]Ga-DOTANOC PET/CT scans were available (overall 268 scans; number of previous PET per pt range: 1–20) and were retrospectively reviewed. Despite the fact that, in most pts, the uptake pattern was stable over time (54/75 pts, 72%), it changed in approximately one third of cases (21/75, 28%). Among DM pts (29/172), only 10/29 (34.4%) presented increased UP uptake. Conclusions: UP [68Ga]Ga-DOTANOC uptake is a frequent non-malignant finding (slightly higher than previously reported), mostly presenting with a diffuse pattern. However, contrary to previous reports, our data show that the pattern of uptake may vary over time in approximately one third of the cases and it is not more frequently observed in pts with DM.

## 1. Introduction

The high expression of somatostatin receptors (SSTR) in neuroendocrine neoplasms (NEN) was exploited to detect well-differentiated tumors (NET) with either somatostatin receptor scintigraphy (SRS) or PET/CT. The use of SRS [1,2] has been largely replaced by PET/CT with beta-emitting somatostatin analogues [68Ga-DOTA0,Tyr3], octreotide ([68Ga]Ga-DOTATOC), [68Ga-DOTA,1-nal3]octreotide ([68Ga]Ga-DOTANOC), and [68Ga-DOTA0,Tyr3]octreotate ([68Ga]Ga-DOTATATE). All these tracers bind the SSTR-2, the predominant receptor type in NEN, and DOTATATE presents the highest binding affinity [3]. DOTATOC and DOTANOC also bind the SSTR-5, and only DOTANOC presents good affinity even for SSTR-3. Despite the reported differences in the receptors’ subtype binding affinity, these radiopharmaceuticals are considered clinically equivalent [3,4,5]. Beta-emitting somatostatin analogues are currently considered the gold-standard imaging procedure to assess patients (pts) with NET and to select those that might benefit from SSTR-targeted therapy with 177Lu and 90Y [5].

The primary indications of [68Ga]Ga-DOTA-conjugate peptide PET/CT include detection of disease extension (staging/restaging determination of SSTR status to evaluate pts who might benefit from PRRT) [5]. However, it is important to note that SSTR are not exclusively expressed on NEN cells; therefore, a careful analysis of patient history is mandatory in order to rule out the presence of non-NEN malignancies [5,6,7] or non-neoplastic disorders [5,8] (e.g., autoimmune diseases, granulomas, infections) that may also show SSTR expression. False positive reporting may also be caused by an erroneous interpretation of the sites of physiological biodistribution [9]. In particular, the presence of increased uptake at the head of the pancreas or at the uncinate process (UP) may represent a pitfall in DOTA-peptide PET/CT image interpretation. Although physiological UP uptake has been described for [68Ga]Ga-DOTATOC, [68Ga]Ga-DOTANOC and [68Ga]Ga DOTATATE, it can be challenging to interpret the findings, especially when a focal pattern is observed, considering that the pancreas is a frequent primary site for NEN. Moreover, the potential misalignment of the PET and the CT both acquired at free breathing may also cause misinterpretation of pancreatic findings [10]. Causative factors at the basis of [68Ga]Ga-DOTA-peptides UP uptake are not fully understood: pancreatic polypeptide (PP) cell hyperplasia has been reported to be associated with UP, and more recently higher frequency of UP was reported in diabetic (DM) pts [11].

The aim of the present study is to evaluate the frequency and variations over time of UP uptake, and to assess whether its presence is more frequently observed in DM pts.

## 2. Materials and Methods

In September 2017, a monocentric prospective observational electronic archive (EC number 131/2017/O/Oss) was initiated at our center, to collect clinical and imaging data of pts undergoing [68Ga]Ga-DOTANOC PET/CT. Among the pts enrolled in the first 6 months (September 2017–February 2018), those presenting with [68Ga]Ga-DOTANOC PET/CT uptake at UP level were further analyzed. Patients with UP lesions already documented on CT/MRI or those that underwent surgical excision of UP before PET/CT were excluded from the analysis. [68Ga]Ga-DOTANOC UP uptake was classified as diffuse or focal and compared with the pattern observed in previous PET/CT scans performed at our center. Increased UP uptake was also correlated with the presence of DM. Data collected in the archive included detailed clinical and morphological/functional imaging history, previous and current co-morbidities (including DM), NEN primary site and grade according to WHO 2010, Ki-67, previous or on-going treatment (e.g., surgical, medical, peptide receptor radionuclide therapy).

[68Ga]Ga-DOTANOC was synthesized by our local radiopharmacy. 68Ga was eluted from a 68Ge/68Ga generator. DOTANOC (ABX) was labelled with the eluted 68Ga, following the procedure described by Zhernosekov et al. [12] and according to the recommendations of the European Association of Nuclear Medicine (EANM) on “good radiopharmacy practice in the preparation of radiopharmaceuticals” [13]. [68Ga]Ga-DOTANOC PET/CT was performed following standard EANM procedure [5] (administered dose: 100–200 MBq; uptake time: 45–90 min after injection) on dedicated cross-calibrated hybrid scanners (Discovery STE; Discovery 710 GE Medical systems, Waukesha, WI) and no specific preparation was requested. PET emission images were recorded 3min/bed position in three-dimensional (3D) modality. CT attenuation-correction acquisition parameters were 120 kV, 80 mA, a 0.8 s tube rotation, and a 3.75 mm slice thickness. Images have been acquired from the skull base to the mid-thigh except in cases where a scan extension was required.

Images were evaluated by four independent expert readers, who assessed the presence of any increased uptake in the UP both visually and semi-quantitatively (standardized uptake value, SUVmax). The [68Ga]Ga-DOTANOC UP uptake pattern was classified as diffuse (when homogeneous uptake involving the UP was present, often elongated longitudinally proximally to the duodenum), focal (intense point-like uptake in the UP without tenuous peripheral uptake) or absent.

The presence of UP uptake was compared with previous PET/CT scans performed at our center (when available) in order to evaluate changes in either presence or pattern of uptake over time. Four categories were identified, in order to describe pattern changes over time:

- focal/diffuse (when the pattern changed/alternated from diffuse to focal);
- focal/absent (when the pattern changed/alternated from focal to absent);
- diffuse/absent (when the pattern changed/alternated from diffuse to absent);
- absent/focal/diffuse (when all the described patterns were observed in the same pt at different time-points).

Increased UP uptake was also correlated with the presence of DM to estimate potential association between frequency of DM and presence of UP uptake.

Mean, standard deviation, median, range, and frequencies were used to describe the data. The chi-squared test was used to assess the correlation between frequencies of UP uptake and the presence of DM.

## 3. Results

From September 2017 to February 2018, 253 pts were enrolled in the archive and 172 out of them were included in the analysis (males: 83; females: 89; median age: 65 ys (range 20–87 ys)). Indications to perform [68Ga]Ga-DOTANOC PET/CT are reported in Table 1. Primary tumor sites are reported in Table 2. In 84/172 (48.8%) cases the primary tumor was excised before PET image acquisition.

**Figure 1:**
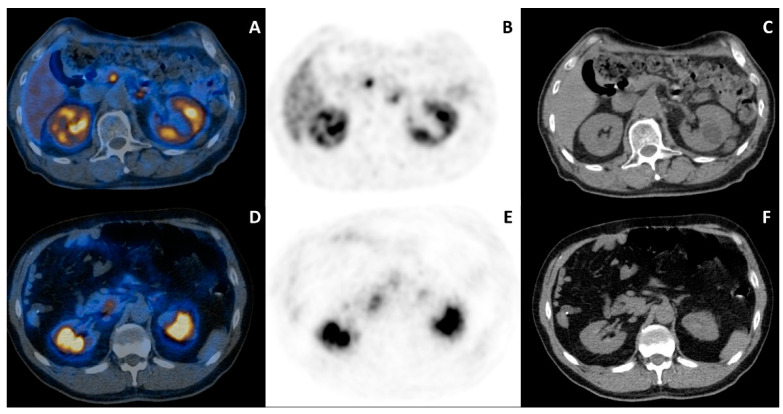
[68Ga]Ga-DOTANOC PET/CT transaxial images (fused PET/CT: (**A**,**D**), PET: (**B**,**E**), low-dose CT: (**C**,**F**)) of two patients showing different patterns of UP uptake. Images of a 76-year-old male pt presenting with a focal UP uptake pattern are shown in (**A**–**C**). Note that the intensity of UP uptake is higher than the adjacent normal liver background. On the contrary, a 57-year-old male presented a diffuse UP uptake pattern (**D**–**F**). The interpretation of a physiologic uptake is much easier when a diffuse pattern is observed.

UP increased uptake was detected in 77/172 (44.8%) pts. Most pts (62/77; 80.6%) presented a diffuse pattern (mean SUVmax = 6.8 ± 2.7 [2.9–13.0]; mean SUVmax of reference liver background = 7.7 ± 2.3 [3.5–13.3]), while a focal one was observed in 15/77 pts (19.4%, mean SUVmax = 6.8 ± 2.9 [3.3–16.4]; mean SUVmax of reference liver background = 7.1 ± 2.4 [3.8–14.0]) (Figure 1).

In order to evaluate whether the evidence of UP uptake persisted over time or if its presence or presentation pattern could vary, previous PET/CT scans of each pt were retrospectively reviewed. Overall, 75/172 pts (43.6%) underwent one or more previous [68Ga]Ga-DOTANOC PET/CT scans at our center. Globally, 268 previous scans were reviewed (2 scans per pt were available in 27 cases; 3 scans per patient were available in the remaining 48 cases. Range of previous PET scans per patient: 1–20; Table 3).

UP uptake pattern was stable over time in most pts (54/75 pts, 72%). In particular, it was always undetectable in 43/75 pts (57.3%), persistently focal in 2/75 (2.7%) and persistently diffuse in 9/75 (12%). However, in almost one third of the studied cases (21/75, 28%), the UP uptake pattern changed over time: focal/diffuse in 5/75 (6.7%), focal/absent in 4/75 (5.3%), diffuse/absent in 11/75 (14.7%), and absent/focal/diffuse in 1 (1.3%), according to the categories defined in the methods section (Table 4).

DM was documented in only 29/172 (16.9%) pts. No blood tests were available to rule out DM in 18/172 (10.5%) pts. Among DM pts, only 10/29 (34.4%) presented UP [68Ga]Ga-DOTANOC increased uptake (focal in 3/10, diffuse in 7/10). Among non-DM pts UP [68Ga]Ga-DOTANOC uptake was evident in 61/125 (48.8%; diffuse: 50/61, 82%; focal: 11/61, 18%). There was no statistically significant correlation between detectable UP uptake and DM (34.4% and 48.8% in DM and non-DM pts respectively, *p* = 0.2).

## 4. Discussion

This report analyses the frequency and the changes in UP uptake pattern over time in a large series of pts with NEN of different primary sites. In particular, the frequency and UP presentation patterns were analyzed in a group of pts scanned at a single center in a short period of time (6 months). Additionally, a retrospective review of previous PET/CT scans was performed (evaluating more than 3 scans in more than half the cases) in order to evaluate possible changes in presence/patterns over time. Overall, our data show that non-malignant [68Ga]Ga-DOTANOC UP uptake can be observed in almost half of the pts, presenting mostly with a diffuse pattern. Our findings also support the fact that UP uptake pattern can vary over time in the same patient in almost one third of cases.

The interpretation of the UP uptake is a well-known issue in [68Ga]Ga-DOTA-peptides reporting: although physiological in most cases, it can be challenging, especially when focal, since the pancreas itself is one of the most frequent NEN primary sites. Although the pancreas can express all the SSTR subtypes on the acinar and islet cellular surface [14], it does not usually show significantly increased [68Ga]Ga-DOTA-peptide uptake [15,16] apart from the UP area.

A description of the frequency of physiological [68Ga]Ga-DOTANOC UP uptake was reported in two previous studies, with slightly lower frequency (31% [17]and 37% [18]). More recently, Lakhotia et al. [19] collected and reported the incidence of physiological uptake described in the literature. They reported an incidence of 47% in the head of the pancreas with [68Ga]Ga-DOTATOC PET/CT [10], 16% in the UP with [68Ga]Ga-DOTATATE PET/CT [20], 70% in the UP with [68Ga]Ga-DOTATOC PET/CT [21], and 26% in the head/UP of the pancreas with [68Ga]Ga-DOTATATE PET/CT [22].

In accordance with previous reports [17], our data confirm that, when present, the UP pattern of uptake is more frequently diffuse. Our data also show that the range of SUVmax is comparable between cases presenting either with a focal or with a diffuse pattern.

Current EANM guidelines [5] recommend careful evaluation of the presence of UP uptake and suggest comparing PET/CT findings with corresponding diagnostic CT images in order to rule out the presence of a morphologically evident lesion at the UP that would require further investigation. During reporting, the assessment of conventional imaging is of primary importance. A normal finding in the region of UP at CT is highly frequent and could help in reporting it as a physiological finding [18,21]. A diffuse UP uptake pattern, coupled with a negative diagnostic CT, is reported as physiologic. This approach based on the correlation between PET/CT and diagnostic CT findings is certainly noteworthy and practical; however, the confidence of the reader is lower when a focal pattern is observed (especially since it is well known and reported in many tumor types that functional changes can precede the evidence of morphological abnormalities) [23]. Finally, the pre-test probability of disease or co-existing genetic syndromes, characterized by a higher susceptibility to develop NEN lesions, may also contribute to a more *defensive* PET reporting attitude.

Factors that contribute to detectable UP uptake are not fully understood. From a pathological point of view, UP uptake was hypothesized to be secondary to pancreatic polypeptide (PP) cell hyperplasia. Wang et al. [24] demonstrated that the UP is very rich in PP cells with a number reduction in α and β cells in this area of the pancreas. PP cell distribution is different in the head region of the pancreas compared to the remaining parenchyma: the majority of the islet cell volume of the head of the pancreas consists of PP cells, while in the remaining pancreatic parenchyma, the contribution of PP cells to islet mass is low. In particular, the restricted area rich in PP cells largely overlaps with the uncinate process with an extension to the nearby parenchyma. It is also worth noting that the head region has distinct properties, anatomically and/or embryologically, that could lead to this region being more prone to be affected in disease states [24]. All these characteristics determine an overlap between physiological and pathological uptake, increasing the interpretation challenge when reporting. The PP cells express SSTR (subtypes 1 to 4) on their surface and can therefore be potentially visualized at somatostatin receptor imaging: two case reports have described a high uptake in UP on 111In-DTPA-octreotide scintigraphy with a histologically proven hyperplasia of the PP cells [25,26]. This may suggest that PP cells are responsible for the uptake in the pancreatic head, as shown in the study by Brabander et al. [11].

However, if the latter was the only causative factor, a stable pattern of the UP uptake would be expected over time.

On the contrary, in our series, in contrast with what was previously reported, in almost one third of the pts, the UP uptake pattern varied over time. In particular, comparing a large number of scans per pt acquired at different time-points, we observed all possible combinations of UP uptake presentation patterns (focal/diffuse, focal/absent, diffuse/absent, absent/focal/diffuse). Therefore, our data do not support previous observation of a permanently stable pattern of UP uptake over time [17]. This finding may probably be explained with the higher number of PET scans reviewed per pt over time. The observation of uptake pattern modifications in some pts suggests that additional factors other than PP-cell hyperplasia alone might be involved to explain transient uptake.

The difference in uptake intensity is probably due to two distinct mechanisms or to an expression of the same mechanism with a different intensity [17]. Castellucci et al. rated a group of 254 scans, related to 100 pts who underwent at least two [68Ga]Ga-DOTANOC PET/CT, and observed that the increased uptake or its absence in the head of the pancreas was stable over time in all pts, due to stability of expression of SSTR types 2, 3, and 5. They considered a prolonged follow-up time (24 months) and the numerous scans examined showed a stable situation in the pancreas, with the absence of biochemical or clinical symptoms. For all these reasons, they hypothesized that the diffuse or focal uptake is likely caused by the physiologic variability in SSTR expression in the pancreas parenchyma and the variability in their anatomic distribution in the organ. Consciousness of these possible and physiological variabilities is very important for a correct interpretation of PET/CT findings [17]. The first study that compared the UP uptake between [68Ga]Ga-DOTATATE SUVmax values and [68Ga]Ga-DOTATOC and [68Ga]Ga-DOTANOC values demonstrated a substantial similarity of SUVmax values among all the tracers examined [20]. Mapelli et al. assessed the SUVmax of physiological [68Ga]Ga-DOTATATE uptake in the pancreas compared with that of suspected findings and tumors: the SUVmax of physiological uptake was lower. They enrolled 100 pts who underwent [68Ga]Ga-DOTATATE for NET of non-pancreatic origin and 38 pts for follow-up of previously treated or suspected pNET [22].

Recently, a potential association with presence of UP uptake and DM was also suggested: Brabander et al. [11] reported a physiological uptake in the UP on 111In-DTPA-octreotide SPECT/CT in 26% of patients. The authors also stated that DM pts have elevated serum PP concentrations, likely due to PP cell hyperplasia [11]. However, elevation of serum PP levels is not specific for DM, but it also happens in infectious or inflammatory disorders [11]. In our series, pts with DM did not show a higher frequency of UP as compared to non-DM cases. It is important to remember that, following the [68Ga]Ga-DOTANOC PET/CT standard EANM procedure [5], a measure of glycemia level before the scans is not mandatory. Reviewing 47 scans, Cueva et al. also did not observe an evident correlation with diabetes or ages [27] in a series of 47 [99 mTc]mTc-HYNIC-TOC scans.

More recently, Oh et al. [28] investigated the association between the physiologic [68Ga]Ga-DOTATOC uptake in the UP and blood glucose level revising a series of 45 scans (44 pts: 22/44 with diagnosed neuroendocrine tumor; 12/44 with diagnosed pheochromocytoma or paraganglioma; 10/44 diagnosed with other tumors except NET including meningioma; one of them underwent two serial [68Ga]Ga-DOTATOC PET/CT scans and both exams were included for the analysis). In fact, PP secretion is stimulated by fasting, exercise, intake of high protein meals and acute hypoglycemia, while it is inhibited by glucose load. The authors suggested that glycemic conditions negatively influence the functional activity of PP cells and the SSTR expression in PP cells, with a consecutively modified interpretation of [68Ga]Ga-DOTATOC images [28]. Watanabe et al. investigated the influence of elevated levels of some hormones on physiologic uptake of [68Ga]Ga-DOTATOC. In particular, they analyzed 167 pts with suspected or known NET, some with elevated levels of ACTH (10/167), gastrin (25/167) or insulin (7/167), and others with normal hormone levels (125/167). A significantly lower physiologic uptake of [68Ga]Ga-DOTATOC in the UP, in the pituitary gland, and in the adrenal glands was found in pts with higher levels of ACTH as compared to pts with normal hormone levels. High levels of glucocorticoid were reported in pts with elevated levels of ACTH, with a decreased expression of SST2 mRNA in pituitary cells, while the expression of SST5 was not altered. This could be the reason to explain the lower uptake in the pituitary gland, while the mechanism in the UP and in the adrenal glands is still unclear (SST2 mRNA expression might be downregulated by high ACTH levels by an unknown mechanism) [29].

## 5. Conclusions

Overall, our data support that an increased [68Ga]Ga-DOTANOC UP uptake can be observed in almost half of the cases. If present, the UP uptake is mostly diffuse; however, in about one third of the cases, a focal pattern can be encountered. In both cases, in the absence of corresponding CT abnormalities, these findings should be interpreted as physiological. Modifications of UP uptake pattern can be observed in almost one third of cases. Our data do not support the observation of a correlation between UP uptake and DM. This observation, coupled with variations in uptake pattern over time in some pts, supports the hypothesis that several and still unknown causative factors are probably involved and could contribute to an increased [68Ga]Ga-DOTANOC uptake at the UP level.

## 6. Pertinent Findings

Among the first 6 months of enrolment of a monocentric prospective observational electronic archive, analyses of a large number of PET/CT scans (n = 268) of NEN pts (n = 172) demonstrated that: UP uptake is a frequent finding (observed in almost half of the cases, slightly higher than previously reported), mostly presenting with a diffuse pattern and, interestingly, characterized by variations over time in almost one third of the cases.

## Figures and Tables

**Table 1:** Indications to perform [68Ga]Ga-DOTANOC PET/CT.

Indication	Number of pts	%
Staging	13	7.6
Restaging after surgery	21	12.2
Before PRRT	1	0.6
Suspected relapse	8	4.7
Restaging after therapy	5	2.9
Interim PET/CT	45	26.2
Unknown primary tumor	13	7.6
Suspicion of NEN	46	26.7
Follow-up	20	11.6
Total	172	100

**Table 2:** Primary tumor site.

Primary Tumor Site		Number of pts	%
GEP			
	Pancreas	33	19.2
	Ileum	48	27.9
	Ileum + duodenum	1	0.6
	Duodenum	1	0.6
	Jejunum	3	1.7
	Appendix	4	2.3
	Colo/rectal	3	1.7
	Gastric	7	4.1
Lung		15	8.7
CUP		18	10.5
MEN		3	1.7
Other	Middle ear	2	1.2
	Kidney	1	0.6
	Skin (merckel cells carcinoma)	1	0.6
	Submandibular gland	1	0.6
	Breast	1	0.6
	Mediastinum	4	2.3
	Paraganglioma	2	1.2
	Adrenals	5	2.9
	Adrenal + lung	1	0.6
	Thyroid	2	1.2
Suspected NEN not confirmed at biopsy/FU *		16	9.3
	Total	172	100

Legend: GEP = gatroenteropancreatic; * = pts studied for suspected NEN without neither bioptical confirmation before PET nor detection of NEN at follow-up.

**Table 3:** Number of previous scans per pt.

Previous Scans Number	Number of pts
1	27
2	13
3	7
4	7
5	7
6	3
7	2
8	1
9	1
10	2
11	3
14	1
20	1
100	75

**Table 4:** Patterns of UP uptake over time.

Pattern		Number of pts%	
Stable			
	Absent	43	57.3
	Focal	2	2.7
	Diffuse	9	12.0
Variable			
	focal/diffuse	5	6.7
	focal/absent	4	5.3
	diffuse/absent	11	14.7
	absent/focal/diffuse	1	1.3
	Total	75	100

## Data Availability

Anonymized data are stored in the Ethical Committee approved eletronic archive at the local investigator site.
